# Traditional vs. Microfluidic Synthesis of ZnO Nanoparticles

**DOI:** 10.3390/ijms24031875

**Published:** 2023-01-18

**Authors:** Maria Leila Popa, Manuela Daniela Preda, Ionela Andreea Neacșu, Alexandru Mihai Grumezescu, Octav Ginghină

**Affiliations:** 1 Department of Science and Engineering of Oxide Materials and Nanomaterials, University Politehnica of Bucharest, 011061 Bucharest, Romania; 2 National Research Center for Micro and Nanomaterials, University Politehnica of Bucharest, 060042 Bucharest, Romania; 3 Research Institute of the University of Bucharest—ICUB, University of Bucharest, 050657 Bucharest, Romania; 4 Academy of Romanian Scientists, Ilfov No. 3, 050044 Bucharest, Romania; 5 Faculty of Medicine, University of Medicine and Pharmacy Carol Davila from Bucharest, 37 Dionisie Lupu Street, District 2, 020021 Bucharest, Romania

**Keywords:** microfluidics, zinc oxide, challenges, nanoparticles, polydimethylsiloxane (PDMS)

## Abstract

Microfluidics provides a precise synthesis of micro-/nanostructures for various applications, including bioengineering and medicine. In this review article, traditional and microfluidic synthesis methods of zinc oxide (ZnO) are compared concerning particle size distribution, morphology, applications, reaction parameters, used reagents, and microfluidic device materials. Challenges of traditional synthesis methods are reviewed in a manner where microfluidic approaches may overcome difficulties related to synthesis precision, bulk materials, and reproducibility.

## 1. Introduction

### 1.1. ZnO

Numerous researchers in chemistry, materials science, physics, medicine, and other scientific disciplines are becoming more interested in studying metal oxides, particularly zinc oxide (ZnO) [1,2,3]. Non-toxicity, high thermal conductivity, high exciton binding energy (60 meV), high electron mobility, and vast bandgap (i.e., 3.2–3.4 eV) are the essential characteristics that make ZnO applicable to an endless variety of applications [4,5,6]. ZnO is receiving significant interest in a wide range of electrical and optoelectronic applications as a material with a direct and broad bandgap. Advantages of a large bandgap include high-power and high-temperature performance, reduced noise production, enhanced breakdown voltages, and the capacity to withstand significant electric fields [7].

ZnO is a semiconductor with either a cubic zinc-blende-type structure or a hexagonal wurtzite-type structure [7,8,9]. As in all solid materials, the atoms of a semiconductor at temperatures above zero are constantly in motion, vibrating about their equilibrium states. The typical material qualities that determine the linear connections between electrical, thermal, and mechanical variables are thermal expansion, pyroelectricity, and specific heat [7]. These thermal characteristics and thermal conductivity are dependent on the surrounding temperature. The highest temperature at which these effects may be researched is the melting point of ZnO, which is 1975 K. Furthermore, because ZnO is commonly utilized in thin-film form deposited on foreign substrates (templates other than ZnO), the characteristics of the ZnO films are intimately dependent on the intrinsic features of the substrates, such as lattice constants and thermal expansion coefficients [7]. Having a melting point of 2242 K (Tm) and a cohesive energy per bond of 7.52 eV, ZnO indicates a high degree of thermal stability [3].

Between 50–60% of ZnO is nowadays utilized in the rubber field, followed by applications in the ceramic industry, concrete fabrication, skin ointments, sunblock cosmetics, nutrition (as a source of zinc), and pigments, with only a small segment devoted to the research of its functional properties. Outside of large-scale manufacturing, many nanomaterials have applications ranging from the electronic industry [10] to biomedicine [11]. ZnO nanostructures (ZONSs) are expanding rapidly in developing applications ranging from biomedicine [12], catalysis, coatings, sensors, and industrial and textiles to energy conversion devices, such as fuel cells and solar cells [13,14].

The methods employed for synthesizing ZONSs play a significant role in their dimensional stability. They can vary from conventional batch synthesis methods used for decades to newer technology platforms such as microfluidics, described in depth in this review article [15]. On a large scale, ZnO may be synthesized as single crystals, epitaxial structures, thin and thick film structures, and nanostructures or nanoparticles. The fabrication techniques are as diverse as the reported structures and applications. Methods are often based primarily on their intrinsic potential and constraints to synthesize a certain sort of structure, while some studies have challenged the conventional limits of growth to synthesize unconventional forms [13].

### 1.2. Microfluidics

Microfluidics is the study and development of systems that process or control extremely small volumes of fluid (on the scale of 10^−9^–10^−18^ L) through channels measuring just tens or hundreds of micrometers in size [16]. It is a science domain that has undergone significant research in recent years, with many devices now able to surpass their classical forebears, as well as the invention of new devices that have permitted the study of processes that were unreachable to macroscale machines [17].

To properly recognize the benefits of these downsized systems, one must first comprehend the physics of fluids that take place on such a size and how this impacts their behavior. Due to the Reynolds Number (Equation (1)), the dynamics of a system may be predicted with more accuracy [17]:

(1) $$Re=\frac{\rho \upsilon L}{\mu }$$

where *ρ* is the density of the fluid, *ν* represents the velocity and *L* is the linear dimension characteristic of the system, and *μ* is the dynamic viscosity.

For on-chip materials synthesis and ZnO applications, the Reynolds number (Re), which provides a ratio of inertial forces to viscous forces, is typically employed to anticipate flow conditions [18].

On the microscale level, these forces outweigh gravity (the dominating force on the macroscale) and may be employed to move fluids without requiring pumps. These factors explain several advantages of microfluidics, including shorter reaction times and easy kinematics. Lastly, the reaction times of microfluidic systems are significantly faster than those of traditional devices. This happens because of the lower system dimensions, which result in a shorter diffusion period for any given molecule [17].

Silicon and glass were used in some of the early fluidic microsystems. Their mechanical stability is highly useful in the emerging area of nanofluidics (the science of fluids in channels with nanometer-scale—ideally less than 50 nm), where channels with hard walls might be advantageous [16]. However, glass and silicon devices are typically unnecessary or unsuitable for studying biological samples in water and have been almost entirely replaced by plastics. Particularly costly and opaque to both visible and ultraviolet radiation, silicon cannot be used in traditional optical detection techniques.

It is simpler to manufacture microanalytical system components, particularly pumps and valves, from elastomers than from hard materials [16]. For most microfluidic device manufacturing techniques, such as photolithography, electron beam lithography, or mechanical engraving, pattern sculpting is required for micro- and nanometer-scale. The procedure involves sketching the design using software applications such as AutoCAD [19].

Since the beginning of the 21st century, poly(dimethylsiloxane), or PDMS, has been the most frequently used and dependable material to manufacture microfluidic chips. PDMS is an optically transparent polymer with excellent flexibility and tensile strength, allowing it to withstand high hydraulic pressure. High-pressure tolerance is an essential characteristic of microfluidic devices, particularly in microfluidic-based nanoparticle synthesis, where the overall flow rate is constantly high for optimal mixing [19]. Although PDMS solidifies during heating, it retains its elasticity. Consequently, a high flow rate may even cause deformation or exposure due to the high pressure within the channels, particularly at the interconnections. To avoid this, the fluidic pressure tolerance is increased by punching holes into the PDMS mold using a needle [19].

Microfluidic technologies have been employed to produce nanoparticles with more control over their physical characteristics to meet the existing issues in nanoparticle formation [20]. The synthetic approaches based on microfluidics provide further benefits such as increased precision, integration capabilities, high flexibility [18], and cost-effectiveness due to decreased raw material consumption, as well as safer handling and storage. Most of the benefits result from shrinking the fluidic environment and the continuous mode of operation [21]. These devices are designed to control the amount of flow and reagents in the microchannels, ensuring that the resulting nanoparticles are uniform despite their low reagent usage [18]. Moreover, multiple process stages and/or parallel reactions can be implemented on a single chip containing microfabricated networks of individually programmable microchannels and reservoirs [22,23].

In the past 10 years, microfluidic chips have proved their incredible potential for providing micro-total analytic systems and merging multiplexed functional units into a single system [18]. Enhanced microfabrication technologies integrate several components into one feasible microelectromechanical system and allow for complex activities, such as regulated continuous and sequential flows, separation, and mixing [24].

## 2. Traditional Synthesis vs. Microfluidics

### 2.1. Traditional Synthesis of ZnO

Traditional nanoparticle synthesis utilizes two fundamental methods: (a) the top-down approach and (b) the bottom-up approach.

#### 2.1.1. Top-Down Approach

**Figure 1:**
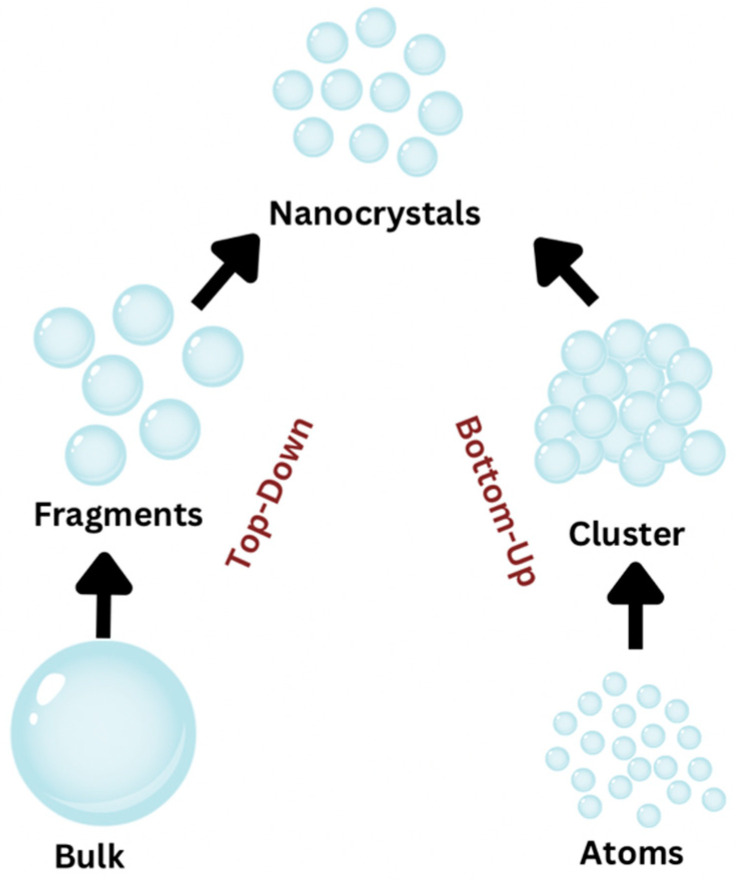
Schematic representation of the two approaches (Top-Down and Bottom-up).

By breaking down bulk material, nanomaterials with particles smaller than 100 nm are produced (Figure 1). An image is projected using lithographic techniques onto a photosensitive emulsion-coated substrate, such as a silicon wafer. X-rays, micromachining, and electron beams are utilized to mill and mold the particles. However, despite their usefulness in manufacturing nanoparticles on a small scale and their remarkable reproducibility, these methods are problematic for large-scale synthesis. In such situations, the bottom-up approach is utilized [25].

#### 2.1.2. Bottom-Up Approach

Nanoparticles are chemically created by assembling molecules and clusters atom by atom until the desired particle shape is achieved [22]. Briefly, “bottom-up” approaches are most frequently employed to create nanoparticles by nucleating and growing particles from small molecule dispersions in liquid or vapor phases [25].

Due to the simplicity with which this approach can be used in industrial production, the bottom-up approach has received considerable attention and is frequently utilized. This strategy provides advantages over the top-down approach, but it also has its disadvantages. Consumption and management of reagents can become problematic [26]. Microfluidic devices have proven to be a relief in this regard because they significantly reduce reagent volume and create nanocrystals with the required characteristics [22].

**Figure 2:**
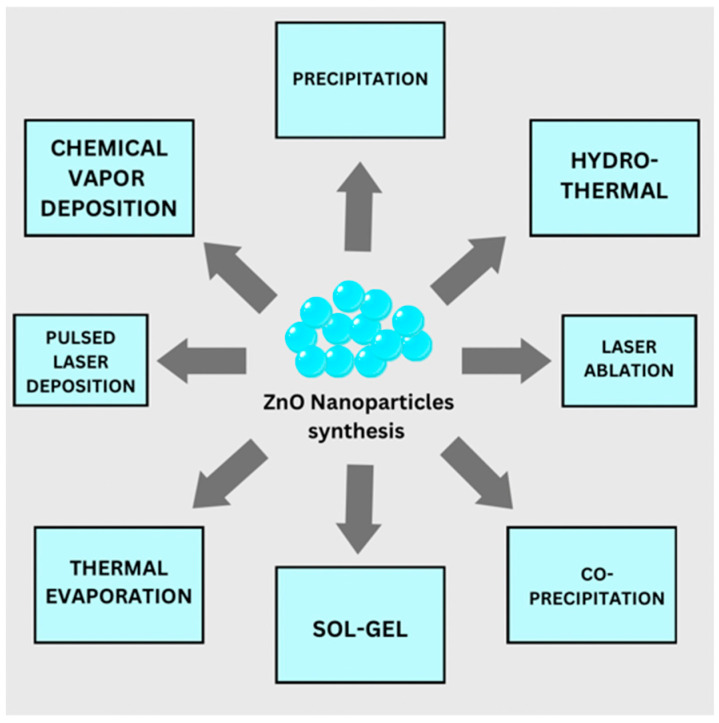
Synthesis methods for ZnO.

ZnO nanostructures are usually synthesized with numerous techniques, such as sol-gel, precipitation, physical vapor deposition, metal-organic chemical vapor deposition, pulsed laser deposition, thermal evaporation, solid-state reaction, and hydrothermal method (Figure 2). These methods are typically chosen based on the requirements and intrinsic capabilities [27].

#### 2.1.3. Hydrothermal Synthesis

Many researchers have been attracted to the hydrothermal approach due to its simple equipment, low cost, and easy preparation. It is an environmentally safe approach. The hydrothermal processing technique can also adjust the size and form of nanomaterials. The reaction time, temperature, and precursor solution concentration primarily determine the morphology of nanoparticles. In turn, this changes the chemical and physical properties of nanoparticles. According to their morphology, particles are selected for wide applications [28]. Besides the hydrothermal synthesis advantages of producing nanometer-sized powders, the morphologies of nanoparticles can be modified by adjusting the reaction conditions, while the prepared powders present different characteristics than the bulk material [29].

**Figure 3:**
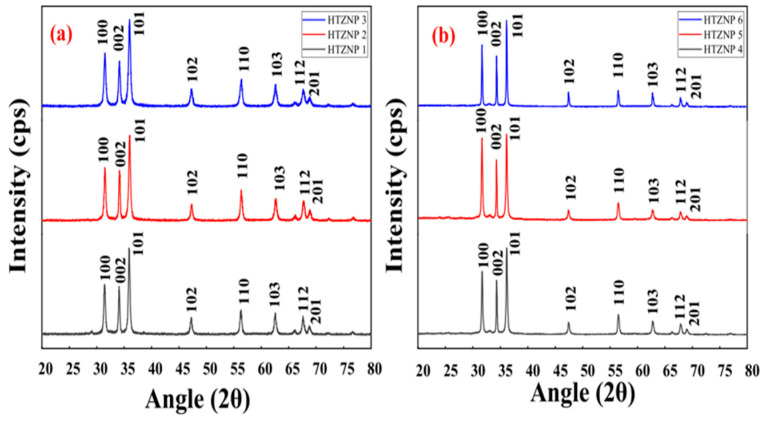
XRD patterns of sample synthesis with constant reaction time and different temperatures (**a**) (HTZNP1, HTZNP2, HTZNP3) and samples with constant temperature and different reaction times (**b**) (HTZNP 4, HTZNP5, HTZNP6) [28]. Reprinted from an open-access source.

Mohan et al. (2020) successfully synthesized ZnO nanoparticles using the hydrothermal technique, either by varying the synthesis temperature (100 °C, 125 °C, and 150 °C) while maintaining time constant or by heating for various periods (120 s, 1.3 h, and 5 h) at a constant temperature (Figure 3). It has been shown that reaction variables play a crucial role in defining nanoparticle size and form. TGA showed that all samples started to degrade at 200 °C and increased to 270 °C as the reaction temperature increased. XRD and TEM investigation indicated that nanomaterial structural characteristics vary with reaction temperature and duration. Therefore, both investigations showed that the sharpness of the diffraction peaks for the samples synthesized at the same temperature is higher than those synthesized at different temperatures, indicating good crystallinity. However, the grain size was found to decrease with an increase in temperature. Hydrothermal synthesis is a good option for producing different nanostructures with significant optical properties. In their study, nanoflowers and nanorods have good photoluminescence, UV absorption, and bandgap. However, nanorods are more stable at high temperatures than nanoflowers [28].

Aneesh and his colleagues (2007) used the same method to synthesize ZnO nanoparticles, but they modified the concentration of the precursors and the temperature. NaOH in the stock solution ranged from 0.2 M to 0.5 M. Structure, grain size, and band gap energy were all influenced by the number of precursors, temperature, and time of growth. The XRD analysis shows that the nanoparticles have a hexagonal wurtzite structure, and the size of the particles grows with the temperature growth and shrinks as the concentration of the precursor increases [29].

The development of ZnO photo-electrocatalysts has been receiving extensive attention in the past years. For example, the large specific surface area that 2D ZnO morphologies possess, such as nanosheets, makes them great candidates for photocatalysis [30].

Mirzaeifard et al. (2020) overcame the challenge of charge carrier recombination of ZnO by doping it with sulfur due to its specific characteristics, similar to the ones of oxygen, for contaminant degradation applications. They synthesized ZnO by hydrothermal and S-doped ZnO by adding thiourea in the same synthesizing method. Both pure and S-doped ZnO showed almost spherical morphology in large particle. The photocatalytic activity was measured in a cylindrical homemade reactor, and most of the S-doped ZnO showed higher photocatalytic activity than ZnO [31].

#### 2.1.4. Laser Ablation

Laser ablation in liquid (LAL) is a sustainable, user-friendly, and effective laboratory technique for manufacturing heterogeneous nanomaterials with varying chemical compositions (particularly metal oxides, sulfides, carbides, and metals) and morphologies. Interest in the laser-assisted synthesis of colloids has increased dramatically over the past decade, which can be attributed to the numerous advantages of LAL, such as reduced amounts (and thus cost) of reagents and solvents utilized, ease of use, and a safe working environment. A laser beam is directed on a metal target immersed in a tiny volume of liquid to ablate the target, generating plasma, vapor, or molten-metal droplets that will further quench and/or react with the liquid to create NPs with various shapes and compositions [32].

Using laser ablation, Mintcheva et al. (2018) fabricated ZnO nanostructures for photocatalyst applications and assessment of shape and effectiveness in organic degradation. ZnO nanospheres (ns-ZnO) and ZnO nanorods (ms-ZnO) were produced by ablation of a Zn metal plate in water utilizing nanosecond- and millisecond-pulsed lasers, correspondingly. The nanorods synthesized by the millisecond laser had far more absorbed water and oxygen molecules, which was assumed to be the reason why they exhibited somewhat better photocatalytic activity than their sphere ZnO counterparts synthesized by the nanosecond-pulsed laser. Thus, the research reveals that laser ablation in liquid environments may be used to synthesize ZnO-based nanomaterials with varying shapes and surface imperfections [32].

#### 2.1.5. Sol-Gel

Sol-gel technique is the most straightforward approach and may optimize particle size and shape by closely monitoring reaction conditions [3]. Some of the sol-gel technique’s most significant benefits are the synthesis’s simplicity, the low temperature of final decomposition, and the ability to modify the chemical composition. This approach has innovative characteristics that are of significant interest because of its low price, ease of preparation, and industrial feasibility [33].

**Figure 4:**
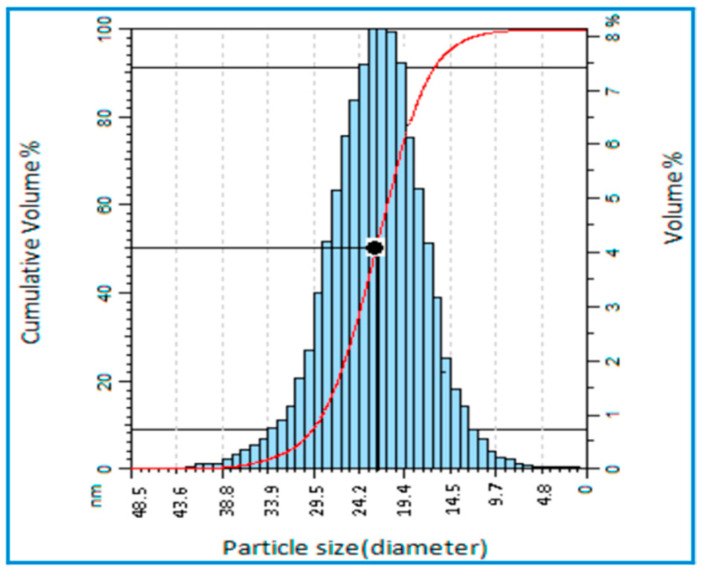
Particle Size Distribution analysis of ZnO shows an almost symmetric distribution at about 22 nm [33]. Reprinted from an open-access source.

Abdullah et al. (2017) synthesized ZnO nanoparticles via the sol-gel technique by combining a methanol solution with zinc acetate dehydrate and then adjusting the pH value of the solution between 9 and 11. This study aimed to manufacture low-dimensional ZnO and investigate its morphological and electrical characteristics. Nano-powdered zinc oxide with an average grain size of less than 50 nm was synthesized using the sol-gel technique, and the powder morphologies were nearly homogeneous. According to the XRD data, a homogeneous, uniform distribution of nanoparticles with an average particle size of 22 nm was obtained. Even though an Atomic Force Microscopy (AFM) test showed that high synthesis temperatures show a high degree of nanoparticle agglomeration, particle size distribution showed as symmetric (Figure 4). Certainly, the greatest benefit of this technique (sol-gel) is its speed and the ability to produce nanoparticle-sized ZnO varistors [33].

Hasnidawani et al. (2016) effectively manufactured ZnO nanoparticles with a nanosize range between 81.28 nm and 84.98 nm using the sol-gel process. Zinc acetate dihydrate (Zn(CH_3_COO)_2_·2H_2_O) was utilized as a precursor, while ethanol was employed as a solvent. Sodium hydroxide (NaOH) and distilled water served as reaction media. The results demonstrated that the ZnO rod-like structure was manufactured using the sol-gel technique in the nanosize range of approximately 84.98 nm. The resulting ZnO nanopowder exhibits excellent crystallinity after synthesis [34].

#### 2.1.6. Co-Precipitation

In contrast to other approaches, the co-precipitation procedure provides a practical way of synthesizing oxide nanoparticles. It results in large-scale production and provides a high-purity nanomaterial using an eco-friendly method without requiring additional high-pressure or high-temperature treatments or dangerous chemical solvents. The growth parameters, such as the kind of precursors, their ratio, and the reaction temperature, make it easy to fine-tune the ZnO characteristics and size distribution [27,35].

**Figure 5:**
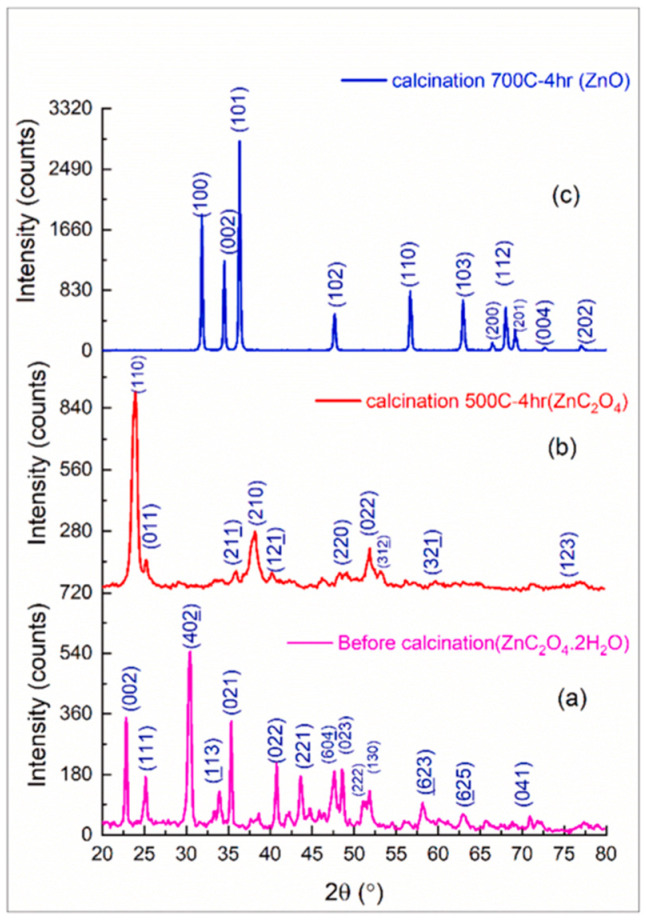
XRD analysis before calcination (**a**), after calcination at 500 °C (**b**), and after calcination at 700 °C (**c**) [36]. Reprinted from an open-access source.

Mahmood et al. (2022) utilized ZnSO_4_·6H_2_O as a zinc supply and oxalic acid as a catalyst. Powdered ZnO nanoparticles were synthesized using the oxalate co-precipitation technique. The calcination technique revealed the transition to zinc oxalate at 500 °C, then to zinc oxide at 700 °C, as indicated by the XRD analysis of samples (Figure 5) [36].

Abdel et al. (2021) prepared ZnO NPs using the co-precipitation technique to study the structure, temperature, and frequency-dependent dielectric and conducting properties. The X-ray diffraction pattern revealed a single-phase wurtzite sample with an average particle size of around 35 nm following TEM imaging. The uniform shape of almost spherical nanoparticles was seen in SEM micrographs. It was discovered that the amplification of the dielectric characteristics of ZnO NPs is attributable to a high number of charge carriers and defect density, which are responsible for rotation direction polarization and space charge. These findings demonstrate the potential of ZnO NPs in creating various electrical devices [27].

#### 2.1.7. Chemical Vapor Deposition

The high-temperature chemical vapor deposition (CVD)-based growth method for high-quality zinc oxide (ZnO) layers provides excellent good control of the growth process and technical simplicity, as well as great crystal quality [37]. To produce high-purity ZnO crystals, films, and powders, gas-phase synthesis techniques like aerosol or chemical vapor deposition (CVD) are favored over liquid-phase precipitation procedures, which are better for controlling particle size. Chemical vapor synthesis (CVS) is a modified CVD method in which nanoparticles created in the gas phase are guided by thermophoresis to a particle collector rather than creating a film [38].

**Figure 6:**
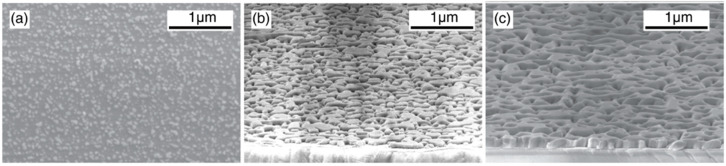
SEM images of the grown samples at 5 min (**a**), 6 min (**b**), and 7 min (**c**). Reprinted with permission from [37]. Copyright (2019) American Chemical Society.

Muller et al. (2019) utilized this strategy to acquire a deeper knowledge of the CVD-based growth methodology. They investigated the formation of ZnO layers on sapphire substrates, also named sacrificial substrate layers, for transparent thin film transistors (TTFTs) and transparent conductive oxides. They controlled a series of samples grown with zinc vapor by controlling the gas methane (CH_4_) precursor to reduce the amount of ZnO powder. Only 10 min of development time was required for ZnO microcrystals to consolidate and create a uniform, closed layer (Figure 6) [37].

**Figure 7:**
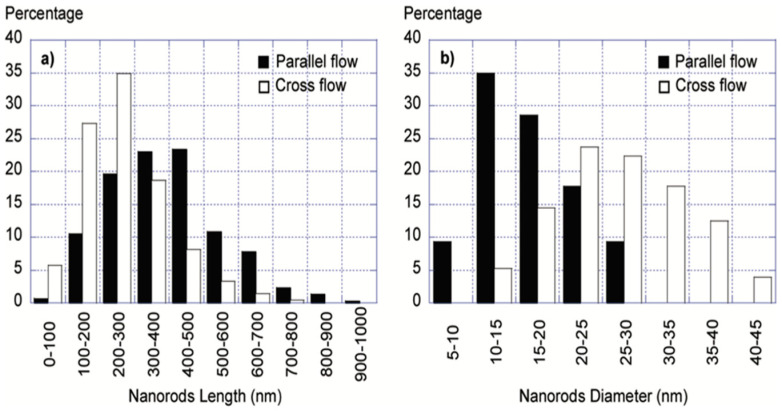
Histogram of nanorods length (**a**) and diameter 9 (**b**) distribution for ZnO nanorods synthesized by parallel flow and crossflow. Reprinted with permission from [38]. Copyright (2009) American Chemical Society.

Reuge et al. (2009) evaluated the effect of key control parameters on the yield and production rate of ZnO tetrapods during CVS from Zn metal precursor by combining experimental fluid dynamics modeling experiments for the first time. The synthesis was conducted in a horizontal quartz tube reactor measuring 110 cm in length and 2.6 cm in diameter, where both parallel flow and crossflow configurations at the same temperature of 900 °C were studied. The mean lengths (250–450 nm) and diameters (14–27 nm) of the nanorods were found to be dependent on the reactor characteristics and flow but not on the air injection location after a total of 860 measurements (Figure 7) [38].

Conventional formulation techniques limit NP translation because they generate large or polydisperse NPs with batch-to-batch variability, resulting in inconclusive findings and processes that are not easily scaled from the discovery process of a study through animal or clinical testing and commercial production. None of the bulk approaches continuously create NPs, resulting in substantial variance across batches [20,39]. Slow heat transfer, nonuniform reactor conditions, especially during injection, and a lack of in-situ observation and feedback impede the control of conventional semiconductor NP synthesis in large-scale batch operations [23].

### 2.2. Microfluidic Synthesis of ZnO

As novel nanoparticle uses emerge daily, nonmanufacturing processes for nanoscale materials that are cost-effective, rapid, and highly repeatable with little environmental impact are urgently required [2]. The greater difficulty is achieving accurate repeatability in each batch. In this context, microfluidic synthesis as a precursor technology for more precise chemical synthesis has garnered considerable attention, particularly in the industrial sector [22].

Unlike conventional batch reactors, microreactors are capable of precise spatial-temporal regulation over reaction conditions such as microchannel dimensions, temperature, pressure, and flow rate, allowing for the continuous synthesis of high-quality ZnO micro-/nanoparticles with well-defined physicochemical characteristics. Microreactors have previously proved the effectiveness of microfluidic techniques in the controlled synthesis of ZnO materials with diverse sizes and forms, ranging from a few nanometers to tens of micrometers [18]. Utilizing microfluidics, nanoparticles (NPs) have been manufactured using mostly common materials, such as poly(methyl methacrylate) (PMMA) substrates or tubes consisting of glass, silicone, or Teflon^®^ [40].

Typically, the production of nanoparticles through microfluidic chips is a one-step process: functional nanoparticles will emerge once the reaction reagents are injected into the inlets. Moreover, due to the regulated flow rates, nanoparticles are of uniform size. For conventional nanoparticle production, neither the “bottom-up” nor “top-down” technique can effectively produce particles of uniform size [19]. Microfluidic devices employ a bottom-up strategy. The bottom-up approach consists of two phases: the nucleation phase and the growth phase. Nucleation is the procedure by which nuclei (seeds) serve as crystal growth templates [41]. In the nucleation phase, precursor particles precipitate in the reaction solution, followed by the growth phase, during which solutes are deposited, and the ideal particle size is formed under the impact of temperature, heat, pressure, and other parameters [22].

**Figure 8:**
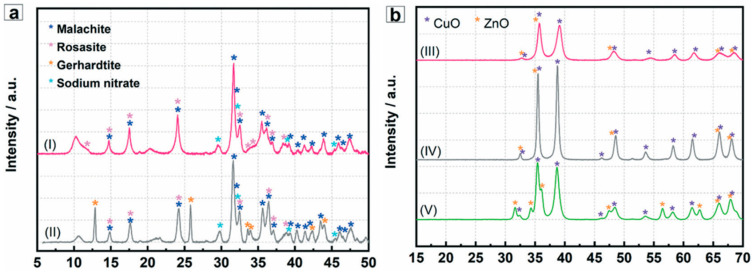
XRD analysis results of uncalcined (**a**) and calcined (**b**) CuZnAl precipitates showing primary precipitates synthesized in microfluidic (I) and batch reactor (II); and microfluidic Cu/ZnO/Al_2_O_3_ (III), batch reactor Cu/ZnO/Al_2_O_3_ (IV) and batch reactor Cu/ZnO (V) [42]. Reprinted from an open access source.

Tofighi et al. (2022) examined a catalytic system for commercial methanol synthesis based on Cu/ZnO/(Al_2_O_3_). They evaluated the system’s analytical results with those of a similar system manufactured using traditional techniques. Compared to catalysts made by batch co-precipitation, the results revealed a rather consistent shape and homogenous distribution of Cu and Zn via the BET test. In addition, XRD examination demonstrated that the development of various phases results from the effective micromixing effect (short-term homogenous mixing) and the spatially regulated nucleation of the primary precipitates in tiny volumes flowing through the microchannel (Figure 8) [42].

#### 2.2.1. Parameters in Microfluidics

The regulated manufacturing of ZnO nanoparticles with well-defined physicochemical characteristics is successful for various shapes [43], including wires, spheres, rods, spindles, ellipsoids, and sheets [44]. Furthermore, microfluidic systems may run continuously and be parallelized to provide greater mass fluxes [45].

Increasing the length of microchannels is a typical method for improving the mixing performance of reactants in ZnO synthesis. Many times, it can be improved by achieving a twisting or winding form. As a result of microfluidics’ uniform reaction environment, the efficiency and operating performance of resulting materials are often greater than those of traditional batch reactors [18].

**Figure 9:**
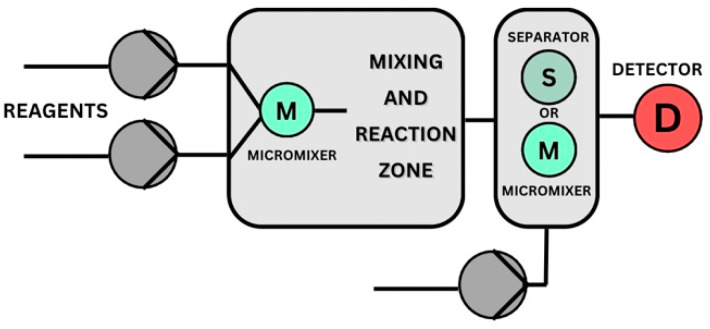
Schematic figure of microfluidic system components.

A simple lab-on-a-chip plate consists briefly of a pump, a micromixer, micro-diameter tubes, and a quench zone (Figure 9) [46].

While syringe pumps are the first choice for small-scale systems requiring continuous operation [47], peristaltic pumps [48] and high-performance liquid chromatography (HPLC) pumps [49] are also good options. Syringe pumps can be found in most microfluidic devices due to the ease of achieving precise and constant flow rates [50]. Furthermore, peristaltic pumps are ideal for dealing with viscous and shear-sensitive fluids [48], whereas HPLC pumps usually run under high pressure, resulting in a greater flow rate than syringe pumps. However, HPLC pumps cannot be utilized with viscous fluids due to the possibility of valve failure [49].

Micromixers are categorized as either active or passive, and the vast majority of micromixers are passive. In an active micromixer, various external energy sources are employed to disrupt the fluid flow, enhance contact area, or cause chaotic advection, hence boosting mixing performance [46,51]. The energy sources can be pressure, electric, magnetic, or thermal field driven [52,53].

In contrast, passive micromixers do not employ any external force to propel the fluids, guide the particles within the fluid or separate them [54]. Passive micromixers are used for their shape and geometry to generate turbulence and chaotic advection of the fluid. Passive micromixers can be classified based on their mixing structures in simple, split, and recombine [55], curved, helical, or multi-layered structures [56].

**Figure 10:**
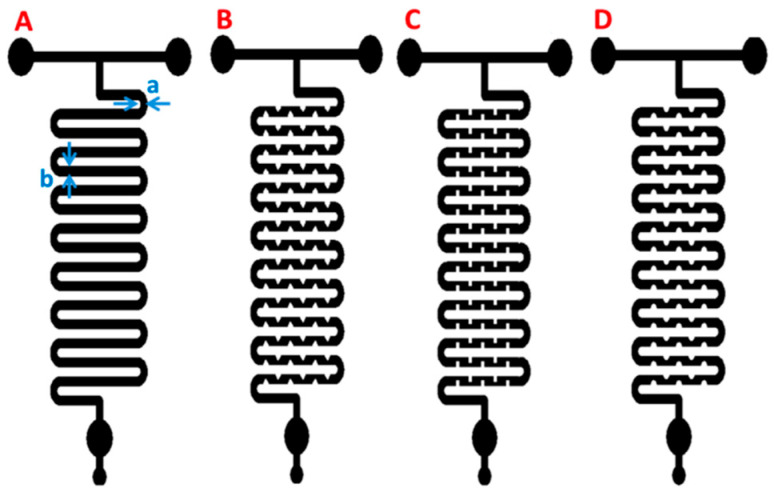
Four different channel geometries for micromixers (**A**) No ridges, (**B**) Triangular ridges, (**C**) Square ridges, (**D**) Smooth/round ridges; a = 200 µm and b = 400 µm [56]. Reprinted from an open-access source.

Passive micromixers have several advantages over active micromixers, including lower cost, greater portability, and simpler integration into Lab-on-a-Chip systems [57]. Moreover, it was found that passive micromixers can be designed to improve mixing in the microfluidic channels. Vijayanandh et al. designed four different types of fluid flow patterns and analyzed them concerning complete mixing and efficiency. They created 3D models and studied them using COMSOL Multiphysics, having ethanol and water as input fluids (Figure 10). The results showed that maximum velocity was present in the center of the microchannels in each case, decreasing when approaching the endings. Triangular-shaped ridges showed the lowest standard deviation, therefore achieving the best mixing of fluids from the four models [56].

Simple structures such as T-type, Y-type [56], or cross micromixers are very easily manufactured; however, they have the lowest performance, split and recombine micromixers are ideal in viscous and multiphase fluids, while helical and curved structures work best at high flow rates [52]. Multi-layered structures have gained a lot of interest lately due to their capability of achieving a great mixing performance in under a few milliseconds while working at both high and low flow rates [57].

Microchannels are usually coil-shaped, rectangular, zig-zag, or even serpentine-shaped [21].

#### 2.2.2. Types of Microfluidic Flow for ZnO Synthesis

In general, microreactors for the synthesis of ZnO materials may be divided into two categories: continuous laminar flow microreactors with single-phase aqueous fluids and two or more inlets for diverse reactants and discrete segmented flow microreactors with one or even more aqueous reactant fluids as inlets but one oil/gas phase for isolating aqueous flows. Due to the microscale size, the flow in microfluidic reactors is almost always laminar. Even though the laminar flow within microfluidic devices makes reactions more regulated, it results in the diffusion-limited mixing of reactants [18].

Continuous laminar flow microreactors: The most prevalent method for the regulated synthesis of ZnO materials within specified microchannels where precursor reactants flow and nucleation/growth processes occur is a single-phase continuous laminar flow synthesis method. This type of microfluidic device has a simpler structure and is easier to operate, allowing for precise control over reaction pressure and temperature, flow rates, residence duration, and reactant concentration [18]. Due to the laminar flow, channel shape governs diffusion length scales, allowing greater control over reagent mixing conditions. In addition, continuous flow microreactors function at a steady state, automatically enhancing reproducibility and enabling real-time system monitoring and feedback [23].

Discrete segmented flow synthesis: Researchers have given very little attention to discrete segmented flow microreactors for synthesizing ZnO nanoparticles. The major emphasis of this microreactor research is the segmented flow between two liquids that forms water-in-oil emulsion droplets. In a microreactor with continuous flow, droplet synthesis of ZnO nanoparticles might also be achieved using microemulsions. Numerous characteristics, such as flow rate, solution viscosity, and microchannel geometry, can influence the production of droplets, which in turn impact the size and shape of ZnO particles [58]. The continuous production of spherical, flower, rod, or even star-shaped ZnO nanoparticles from discrete flow microreactors was possible by selecting the appropriate precursor and reactant concentrations [18].

## 3. Traditional vs. Microfluidic Synthesis of ZnO Morphology

### 3.1. ZnO Morphology in the Traditional Synthesis

#### 3.1.1. Wire/Rod

**Figure 11:**
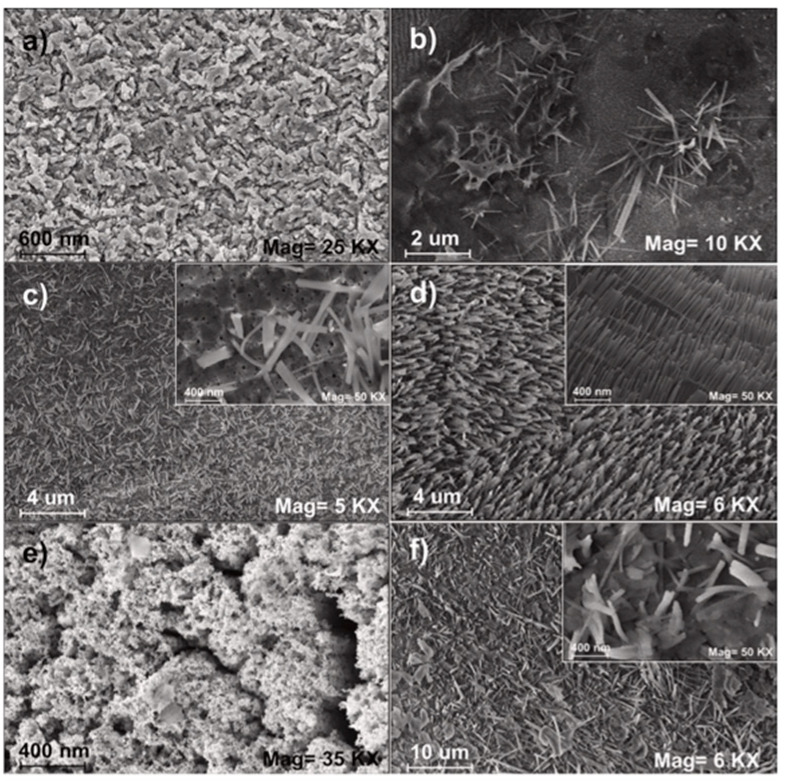
FE-SEM micrographs of ZnO nanostructures at applying voltages of (**a**,**b**) 40 V, (**c**,**d**) 30 V, and (**e**,**f**) 20 V. Reprinted with permission from [59]. Copyright (2021) Elsevier B.V.

Tello et al. (2021) described the synthesis of ZnO nanowires employing an untested electrochemical anodization method using an electrolyte medium mostly composed of ethylene glycol and fluoride ions. Field emission scanning electron microscopy (FESEM) and transmission electron microscopy (TEM) measurements reveal a nanowire shape with an average length of 296 nm. However, by applying different voltages of 40 V, 30 V, and 20 V, they produced distinct morphologies such as nanoflakes (Figure 11) with average size values of about 160 nm and nanowires having an average length of 383 nm and sponge-shaped nanoparticles, respectively (Table 1) [59]. 

**Figure 12:**
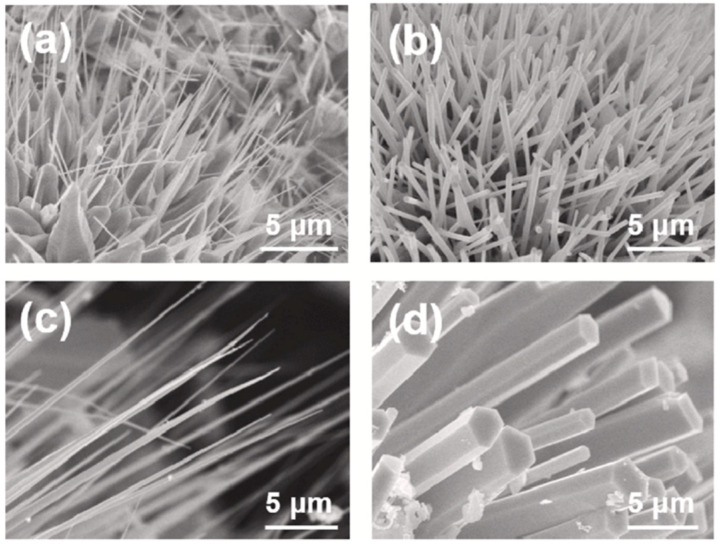
SEM Micrographs of ZnO nanowires at a different microwave input power of (**a**) 1 kW 0 slm, (**b**) 1.5 kW 0 slm, (**c**) 1 kW 3 slm and (**d**) 1.5 kW 3 slm. Reprinted with permission from [66]. Copyright (2021) Elsevier B.V.

Lee et al. (2021) synthesized ZnO nanowires using microwave plasma at atmospheric pressure by adjusting the microwave source power and water vapor to regulate the nanowires’ sizes and lengths. The microwave input power correlates with plasma temperature, whereas the introduction of water vapor correlates with OH radical concentrations as an oxygen source. The ZnO nanowire width grew as the microwave input power increased (Figure 12), but the nanowire length grew when water was added. Consequently, it was established that the diameters and lengths of ZnO nanowires can be regulated by altering the input power and the quantity of water supplied by the microwave plasma at atmospheric pressure. With the addition of water, the length of the nanowire expanded as the input power increased [66].

#### 3.1.2. Sphere

**Figure 13:**
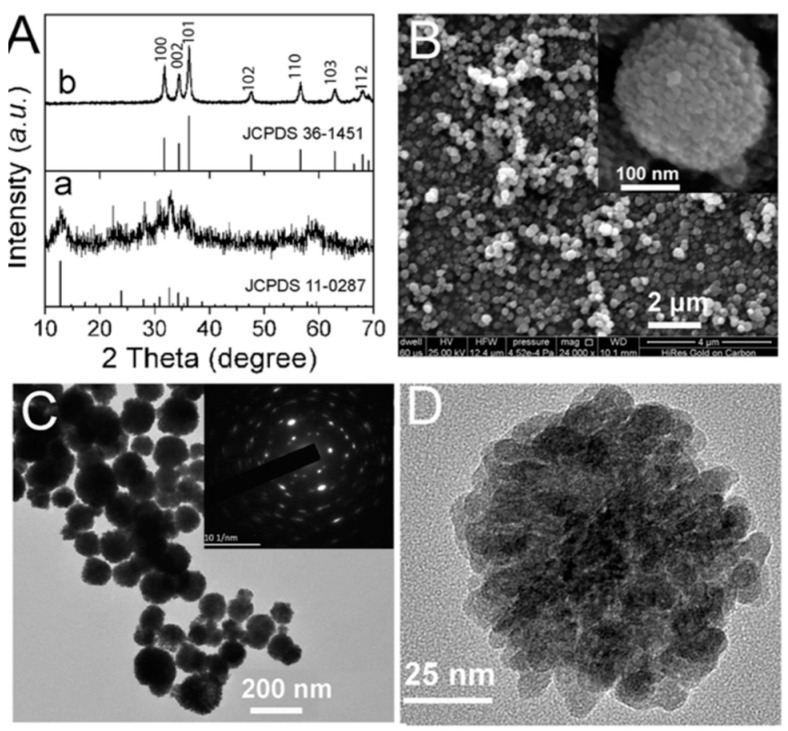
XRD analysis of ZHCH (a) and ZnO (b) (**A**) and SEM micrographs of ZnO showing agglomeration of small nanoparticles that compose nanospheres (**B**–**D**). Reprinted with permission from [60]. Copyright (2017) Elsevier B.V.

Tian et al. (2017) used a thermal treatment technique to synthesize mesoporous ZnO nanospheres. The ZnO nanospheres were manufactured by thermally decomposing the zinc hydroxide carbonate hydrate (ZHCH) precursor using diethylene glycol (DEG). XRD diffraction patterns demonstrated that the precursor ZHCH degraded into ZnO nanoparticles (Figure 13), whilst SEM and TEM investigations revealed mesoporous ZnO nanospheres. In addition, several ZnO nanospheres looked composed of many nanoparticles with lower sizes (around 20 nm) [60].

Zhang et al. (2011) synthesized ZnO nanospheres using a hydrothermal and wet-chemical approach and compared the obtained results in terms of morphology. Each particle had a normal spherical form, but the diameter varied according to the production method. The nanospheres synthesized by the hydrothermal approach ranged in size from tens to hundreds of nanometers. Still, the SEM examination revealed that the nanospheres generated by the wet-chemical method could not be detected because of the presence of organic molecules. The hydrothermal approach seemed more effective in producing nanoparticles with a sphere shape and a nice crystal structure [61].

Li et al. (2011) investigated the micro-continuous flow synthesis of diverse ZnO microparticle morphologies. If surfactants, polymer additives, or other water/solvent mixes are utilized, the spectrum of particle morphologies can be expanded further. Small spheres (about 150 nm in diameter) were generated in 1% polyacrylamide, whereas bigger spherical particles were created in a 1:1 combination of water and isopropanol [67].

#### 3.1.3. Flower

**Figure 14:**
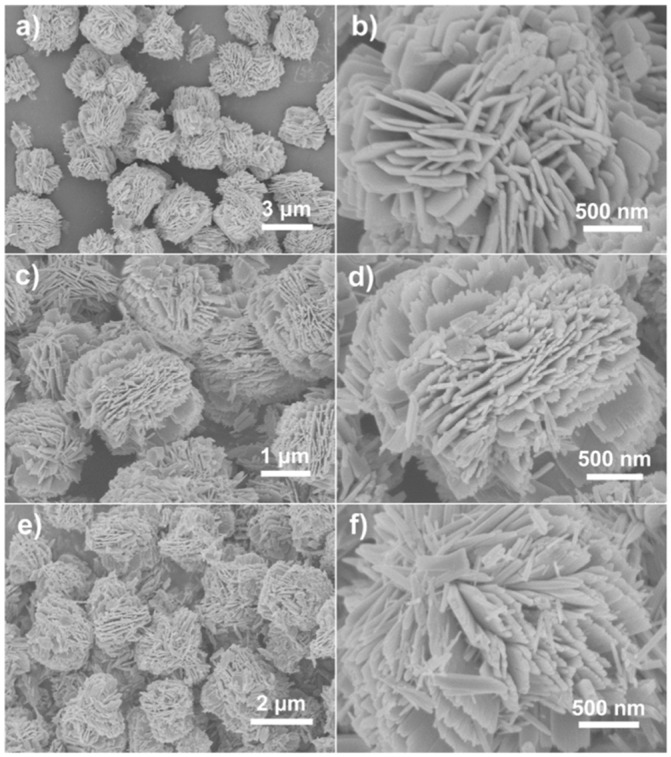
SEM micrographs of directly hydrothermal synthesized ZnO nanoflower (**a**,**b**), and low (**c**,**d**) and high (**e**,**f**) ultrasonic treatment synthesized ZnO nanoflowers. Reprinted with permission from [62]. Copyright (2020) Elsevier B.V.

Qu et al. (2020) utilized a straightforward ultrasonic treatment to regulate the manufacture of three distinct types of 3D fluffy ZnO nanoflowers having distinct nanostructures. Specifically, one nanoflower with smooth edges was generated by direct hydrothermal reaction without ultrasonic treatment. In contrast, the other two nanoflowers with distinct jagged edges were achieved by ultrasonic treatment at different power before the hydrothermal method (Figure 14). With increasing ultrasonic intensity, the size, specific surface area, crystallite dimension, inherent donor defects, and signals of reactive radicals of ZnO nanoflowers decreased. According to the imaging data, the edges of the nanosheets in the initial ZnO nanoflower were smooth, but the edges of the nanosheets in the other two nanoflowers were jagged [62].

Tripathi et al. (2014) established an environmentally friendly method for producing ZnO nanoflowers from *Bacillus licheniformis* with the purpose of photocatalytic applications. After drying a mixture of zinc acetate dihydrate, sodium bicarbonate, and bacterial biomass, ZnO nanoflowers were found. The results indicated that the ZnO nanoflowers were between 250 nm to 1 m in size. In addition, the data revealed nanopetals of approximately 40 nm in width and 400 nm in length [63].

#### 3.1.4. Sheet/Flake

**Figure 15:**
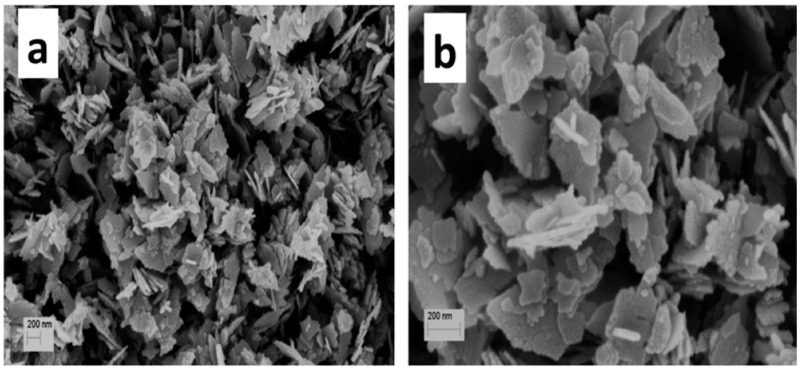
SEM micrographs showing the formation of the synthesized ZnO nanoflakes; (**a**,**b**) different magnification. Reprinted with permission from [64]. Copyright (2015) Elsevier B.V.

Samanta et al. (2015) successfully employed a simple wet chemical process to produce ZnO nanoflakes. Under continuous stirring, zinc chloride (ZnCl_2_) and sodium hydroxide were combined drop-by-drop to produce nanoflakes. FESEM scans demonstrated that this synthesis process was successful in producing nanoflakes with diameters of around 200 nm and a thickness of 30 nm. In addition, the SEM analysis revealed that the Zn(OH)_2_ nuclei produced by the wet chemical reaction expanded in response to the temperature, resulting in the formation of nanoflakes (Figure 15) [64].

#### 3.1.5. Ellipsoid

**Figure 16:**
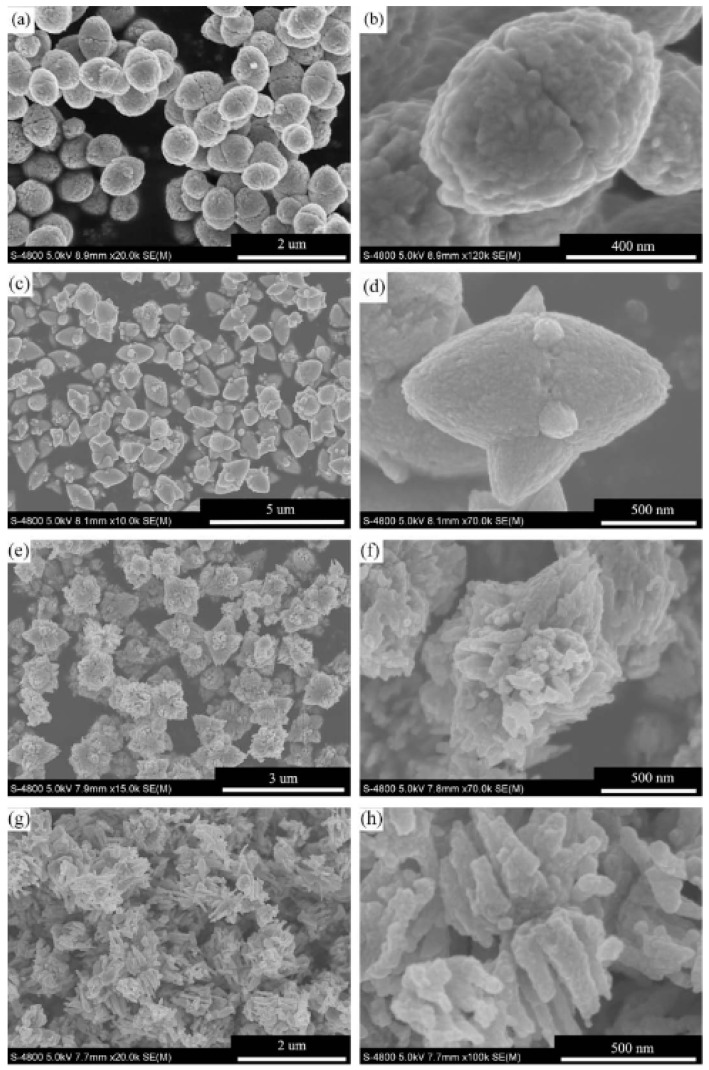
SEM micrographs of ZnO synthesized at pH values of 6 (**a**,**b**), 8 (**c**,**d**), 10 (**e**,**f**), and 12 (**g**,**h**). Reprinted with permission from [65]. Copyright (2010) Elsevier B.V.

Pu et al. (2010) synthesized ZnO ellipsoidal nanostructures quickly utilizing a chemical synthesis process at 90 °C without using other procedures such as calcination, sonication or laser, or organic additions. End-to-end oriented attachment along the major axis and side-by-side oriented-attachment along the minor axis are the factors that contribute to the self-assembly of ellipsoidal nanostructures. Sequentially, two half-ellipsoids of ZnO nanostructures germinate. In addition, they studied the shape evolution of ZnO ellipsoidal nanostructures as the solution’s Zn^2+^ concentration and pH values changed (Figure 16). They observed that these variables substantially influenced ZnO nanostructure morphologies [65].

Duan et al. (2007) combined ZnO nanorods into ellipsoid-like ZnO structures. They utilized a method of self-assembly of nanoscaled building components. In this particular instance, they utilized an autoclave test and a pyrolysis-integrated approach to produce ZnO ellipsoid-like structures made of nanorods undergoing an oriented-attachment process. They have found that raising the pressure in the autoclave reduces the size of the nanorods and strengthens the structural integrity. They developed ellipsoid-like nanostructures with a short axis between 200 and 300 nm and a long axis between 300 and 500 nm [68].

### 3.2. ZnO Morphology Microfluidic Synthesis

The nucleation, development, and creation of well-defined ZnO micro-/nanoparticles require a significant amount of time (at least several hours or days) with most of these approaches. Accurate control of the nucleation stage throughout the synthesis process is also a significant obstacle for these conventional batch procedures, making them more susceptible to low repeatability [69].

For instance, Hao et al. (2019) have developed an efficient microfluidic flow system for synthesizing ZnO nanostructures, demonstrating that by adjusting the flow rates and reactant concentrations of the two inlet fluids in their spiral-shaped microreactor, they were able to obtain seven different shapes of ZnO structures [69].

**Figure 17:**
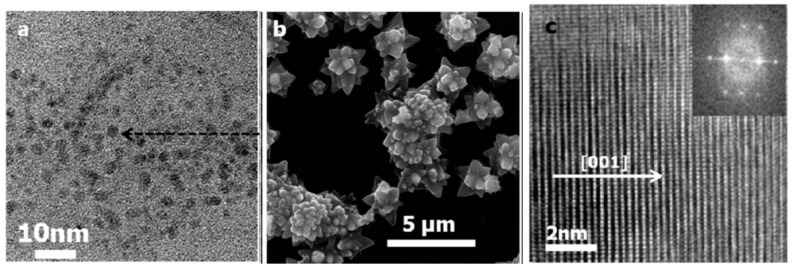
SEM micrographs of (**a**) spherical ZnO NPs, (**b**) flower-like ZnO NPs, and (**c**) ZnO Nanowires. Reprinted with permission from [70]. Copyright (2014) American Chemical Society.

Choi et al. (2014) utilized a continuous flow microreactor system with the same solutions while altering the flow rate and rotations per minute (RPM) to create crystalline ZnO films, vertical ZnO nanowires, flower-like ZnO films, and ZnO nanowires (Figure 17). They observed that the deposition duration substantially impacted the thickness and size (Table 2) [70].

#### 3.2.1. Wire/Rod

Among all the ZnO particle forms produced by microreactors, the wire/rod shape has gotten the most attention from scientists. Typically, the flow synthesis of ZnO wires/rods is usually accomplished via in situ deposition onto supporting substrates within microchannels to create ordered arrays.

Kim et al. (2012) have experimented with nanowire-integrated microfluidic devices to study cell lysis. The cells have successfully traversed ZnO nanowired microfluidic channels, with their performance being greatly impacted by the nano and micro/nano hybrid structure arrangement [71].

**Figure 18:**
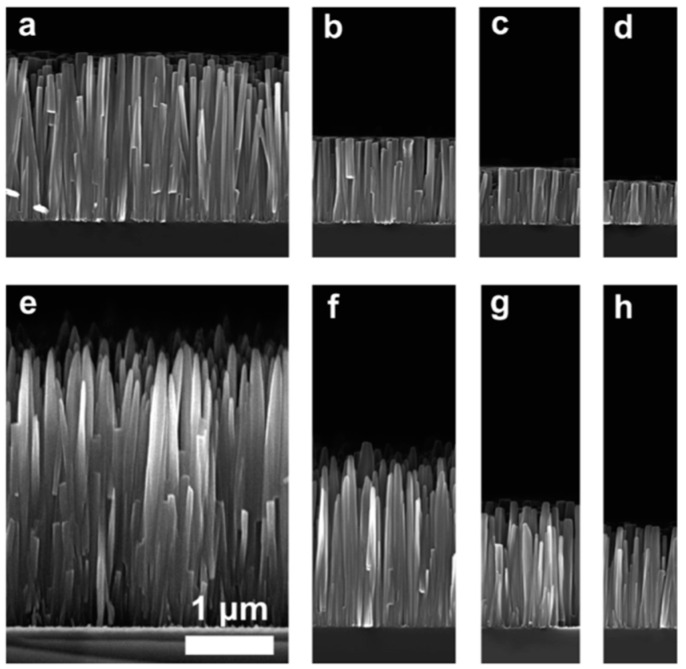
SEM micrographs showing cross-section images of the ZnO nanowires at low flow rates (**a**–**d**) and high flow rates (**e**–**h**). Reprinted with permission from [72]. Copyright (2009) American Chemical Society.

McPeak et al. (2009) have also investigated the chemical deposition of ZnO nanowires in a continuous-flow microreactor with a submillimeter tunnel on a glass/silicon substrate serving as the reactor wall. Deposition at high flow rates leads to more homogeneous nanowire arrays, while deposition at low flow rates results in greater diversity in nanowire length throughout the substrate (Figure 18) [72].

#### 3.2.2. Sphere

The range of potential particle sizes for sphere-shaped nanoparticles generated using microfluidics techniques was limited to just a few tens of nanometers. Continuous synthesis of ZnO spheres is possible using laminar flow microreactors or even more complex integrated microfluidic devices [18].

Stolzenburg et al. (2018) investigated the benefit of particle creation by utilizing a tiny amount of material in microfluidics. At increasing temperatures, they discovered that the reaction mixture diffuses into the surrounding fluid, causing the droplets to shrink and finally assemble into highly spherical aggregate forms. Additionally, they evaluated several plate designs for microfluidic devices. They observed that ZnO synthesis at lower temperatures may be accomplished depending on the model system without droplet shrinking, resulting in 150 nm aggregates of arbitrary shapes that self-assembled inside the microreactor. In addition, ZnO nanoparticle production during nonaqueous synthesis happens quicker and at lower temperatures than TiO_2_ synthesis [45].

#### 3.2.3. Flower

In many studies, it was the chemical precursors that determined the morphology, but in the continuous flow microreactor system, different morphologies can be obtained by adapting physical parameters such as the flow rate and the RPM of the rotating disk, while maintaining the same chemical precursors and precursor solution concentrations.

Choi et al. (2014) investigated the aqueous production of ZnO nanocrystals, nanoassemblies, and thin films by altering merely physical factors. Only powdered flower-like ZnO were formed using a batch reactor under the same experimental parameters but two distinct flow rates of 14.7 and 28.1 mL/min, whereas the deposition duration impacted the other three film forms. However, flower-shaped ZnO microcrystals at a lower flow rate looked bigger, while those generated at a greater flow rate appeared to have more petals [70].

Kim et al. (2013) effectively produced Na-doped flower-shaped ZnO microparticles having efficient photocatalytic activity in a continuous flow microreactor. The continuous synthesis of ZnO flowers was performed in microreactors with laminar flow and a T-shaped mixer [73].

#### 3.2.4. Sheet/Flake

Two comparable methods may accomplish microfluidic production of ZnO sheet/flake nanostructure: in situ growth technique and ex-situ generation technique [18,76].

**Figure 19:**
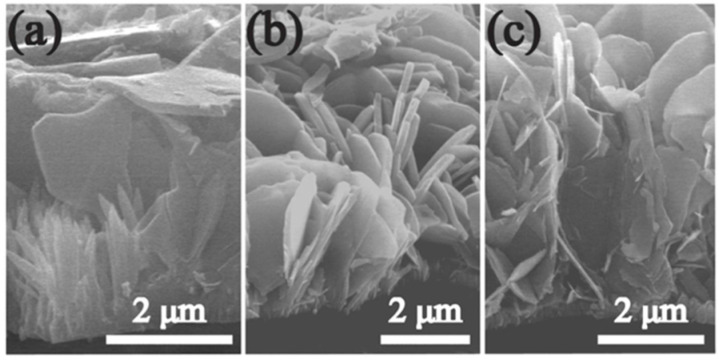
SEM micrography of the ZnO nanostructures side view where both nanoflakes and nanorods can be identified in the same structure. Side-view SEM images of represented AZO nanostructures (**a**) AZ1, (**b**) AZ2 and (**c**) AZ4. [77]. Reprinted from an open-access source.

Chao et al. (2015) investigated an in-situ synthesis procedure that was demonstrated to be an effective and scalable method for synthesizing nanostructures with controlled properties. The morphology was modified by using a microfluidic control unit to regulate the number of additives. This resulted due to the fact that reagents were directly delivered to the reaction area, and therefore, they obtained both nanoflakes and nanorods mixed in the same structure (Figure 19) [77].

#### 3.2.5. Ellipsoid

Choi et al. (2013) investigated ZnO nanocrystals’ development process and stability by manipulating an aqueous solution’s pH and flow parameters. In order to generate three-dimensional mesoporous ZnO assemblies with ellipsoid and spherical forms, electrostatic interaction and Dean vortices interactions were utilized. However, continuous laminar flow synthesis of ellipsoid-like ZnO materials may normally be accomplished by a self-assembly technique [78].

## 4. Applications of ZnO Synthesized through Microfluidics

Due to its outstanding characteristics and many prospective applications in electronics [79], energy [80], biomedical engineering, drug delivery [81], and catalysis [82], zinc oxide (ZnO) micro-/nanomaterials have drawn the interest of materials scientists and engineers.

The development of microfluidics introduces several novel and desirable characteristics that conventional batch systems are unable to attain, hence creating new application opportunities.

Similarly, from the perspective of downstream applications, microchips based on microfluidics might provide several advantages over standard batch methods, such as high specificity and sensitivity, quick reaction time, simple sample preparation, and reduced sample consumption. These characteristics equip microchips with applicability in sensing [83], nanomedicine [84], drug delivery [85], tissue engineering [86], cell cultures [87], and several other biomedical domains [88].

The fast development of microfabrication methods has resulted in the development of microfluidic reactors for ZnO material production and microfluidic devices for on-chip applications [18].

Microfluidic device fabrication has historically depended heavily on semiconductor and microelectromechanical systems (MEMS) industry-derived micromachining technology. These methodologies have enabled the fabrication of microfluidic platforms with fully-integrated microelectronics—a necessity for applications including electrophoresis and dielectrophoresis (DEP), surface acoustic wave (SAW) actuation, digital microfluidics, and on-chip electrochemical detection [89].

### 4.1. Immunofluorescence Sensing

Nanostructure-enhanced detection is the potential for various applications, including early cancer diagnosis, environmental monitoring, and mining safety. Nanostructure-integrated microfluidic chips offer the unique benefit of ultra-low quantitative analysis [74,90].

Guo et al. (2018) successfully generated dense ZnO nanowires of varying diameter and length for protein detection by varying the polyethyleneimine (PEI) concentration and growing hydrothermal period in microfluidic channels. Due to the flow-induced replacement of nutrients and the effect of shear stress, the strategy was more effective than the usual hydrothermal technique. In addition, they utilized the improved ZnO nanowires to exhibit various cancer biomarker detection. The results indicated that ZnO nanowire-integrated microfluidic devices hold promise for high-throughput fluorescence-based diagnostic tests [74].

### 4.2. Electrochemical Sensing

In recent years, novel detection techniques such as fluorescence nanoparticle probes, nanostructured devices, and surface-enhanced Raman spectroscopy (SERS) have been developed [91,92].

Label-free sensing detection of analytes in low-volume, highly diluted, and multicomponent mixtures is crucial in various disciplines. Surface-enhanced Raman scattering (SERS) technology facilitates the direct, sensitive, nondestructive, and real-time capture of quantitative chemical information, whilst the microfluidic approach enables the precise manipulation of small doses of fluids. Therefore, the combined microfluidics-SERS platform is a promising technique for label-free analysis of a small quantity of analytes [18].

Nanostructures based on SERS and ZnO can be utilized as probes for cell detection, intracellular investigation, and imaging. Using SERS detection to identify normal cells from malignant cells, for instance, might be a good application. Concerning protein and DNA detection, Xie et al. (2014) examined the ability of 3D Ag-coated ZnO (Ag/ZnO) substrates to detect the HeLa cell spectral signature. Combined with such a microfluidic system, this substrate was designed to trap cells in horseshoe-shaped formations, therefore bringing cells physically close to the substrate. They evaluated the SERS sensitivity of the nanostructures by detecting a 4-amino thiophenol (4-ATP) molecule, and strong signals were observed in the case of AgZnO substrates compared to no signals from simple Au film or ZnO nanorods [93].

### 4.3. Biological Separation

Li et al. (2017) present a unique approach for isolating and releasing circulating tumor cells (CTC) utilizing a simple wedge-shaped microfluidic device embedded with a substrate containing degradable zinc oxide nanorods (ZnNRs). Several methods have been employed during the past decade to overcome the difficulty of identifying and isolating CTCs in blood samples from cancer patients. These approaches consider several methods for cell enrichment, including microfluidic technology. Due to the increased frictional resistance between cellular surface components and the substrate containing nanostructures, the capture efficiency of microchips with ZnNRs substrate was greater than that of microchips with pure glass substrate [94].

### 4.4. Photocatalysts and Photodegradation

The unique features of ZnO, which may be utilized in dye-sensitized solar cells and photocatalysts, have drawn increasing attention over the past several decades.

Han et al. (2013) investigated a microfluidic device with incorporated ZnO nanowires for organic dye photodegradation research. They considered immobilizing the photocatalysts on a solid surface because previous research indicated that catalysts such as TiO_2_ nanoparticles (NPs) spread uniformly in an aqueous solution for the photodegradation of organic pollutants under Ultraviolet light is not an effective method due to limited light coupling efficiency.

ZnO nanowires have a faster electron transfer rate than TiO_2_ NPs. They can be easily manufactured on a solid surface, making them a viable option that might be incorporated into a microfluidic device.

The objective of their research was to establish a bench procedure for integrating ZnO nanowires synthesized using hydrothermal synthesis into microfluidic devices. Notable also is that under ideal circumstances, the photodegradation rate of microfluidic device-integrated ZnO NWs is significantly higher than that of bulk conditions [95].

**Figure 20:**
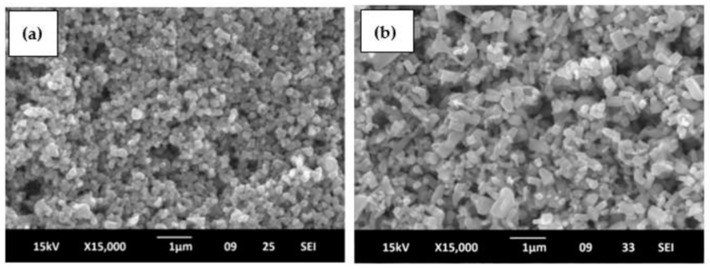
SEM showing TiO_2_ (**a**) and ZnO (**b**) morphologies [96]. Reprinted from an open-access source.

Mohamad et al. (2022) proved that ZnO is an excellent alternative in dye-sensitized solar cell (DSSC) devices due to its high electron mobility. They compared a DSSC device having ZnO as a photoanode cop test cell with a DSSC device that uses TiO_2_ and Ruthenium N719, proving several limitations of the latter, such as low electron mobility, the metastability of TiO_2_, and the high cost of Ru N719. Both TiO_2_ and ZnO were synthesized chemically and deposited on the photoanode, which was further layered with three different organic dyes. Furthermore, the energy conversion and properties were assessed in a solar simulator at 1000 W/m^2^ of light, and then the surface morphology and properties were examined using X-ray diffraction, SEM, and UV spectroscopy. The ZnO DSSC showed better open circuit voltage and electrical performance, confirmed by the rectangular nanostructure with a higher surface area than the TiO_2_ (Figure 20) [96].

## 5. Drawbacks and Current Challenges

### 5.1. Microfluidic Synthesis Drawbacks

Unfortunately, only a limited number of 3D printing methods can achieve the small feature sizes required by many microfluidic applications. In addition, the declared resolution parameters of a 3D printer rarely correspond to the possible feature size, a difference caused by both the machine’s mechanical control and the printing material. The inability of 3D-printed devices to sometimes maintain the same secondary structures or physical surface features, such as permeability and biocompatibility, required for their application is still a challenge [97].

### 5.2. Current Challenges

The current techniques for manufacturing microfluidic devices include micro-milling, injection molding, micromachining, and hot embossing. However, these procedures may be time-consuming, inaccurate, costly, and difficult to modify. Conventional microfluidic devices also lack true three-dimensional (3D) architecture and device design flexibility. Lastly, the generally utilized materials for fabricating microfluidic devices may still have disadvantages, such as surface adsorption and solvent swelling for polydimethylsiloxane (PDMS) or gas impermeability and stiffness of thermoplastics such as polymethyl methacrylate (PMMA). 3D printing has lately gained popularity as a technology for producing microdevices because it offers various benefits over conventional fabrication methods [97].

Fused deposition modeling (FDM) tends to be the least expensive 3D-printing option to acquire and maintain because of the printer’s materials and its cheap manufacturing cost. Nevertheless, there are a few examples of 3D printing being utilized to produce microfluidics reactors for the synthesis of nanoparticles [40].

## 6. Conclusions

In conclusion, microfluidics provides a novel technology system for nanomaterial synthesis and molecule manipulation, accelerating development in various fields such as biomedicine, chemistry, and materials science. This review provides relevant examples that support the merit and the advantages of using such an automated microsystem in multiple situations. In the past decades, microfluidic platforms have not only emerged, but they have rapidly expanded as great alternatives to chemical synthesis. This review is designed in such a manner to provide insights and strategic guidance on different types of ZnO NPs synthesis with respect to the conventional batch synthesis and morphology. Microfluidics provides in-depth differences in the field of research by overcoming the difficulty of complex and stepwise methods.

## Figures and Tables

**Table 1:** Synthesis of ZnO nanoparticles via traditional methods.

Method	Materials	Size	Shape	Reference
Electrochemical anodization	Ethylene glycol, fluoride ions	383 nm	Wires	[59]
Microwave plasma	Zinc powder, Oxygen, and Nitrogen gas	10–340 nm	Wires	[47]
Thermal treatment	Zinc hydroxide carbonate hydrate, Diethylene glycol	20 nm	Sphere	[60]
Hydrothermal method	Zinc acetate dihydrate, Diethylene glycol	10–20 nm	Sphere	[61]
Ultrasonic treatment	Zinc nitrate, Sodium hydroxide, Hexamethylenetetramine (HTMA)	270–350 nm length, 18–40 nm width	Flower	[62]
Biogenic synthesis	Zinc acetdihydrate, Sodium bicarbonate, Bacterial biomass (Bacillus licheniformis)	400 nm length, 40 nm width	Flower	[63]
Wet Chemical method	Zinc Chloride, Sodium hydroxide	200 nm diameter, 30 nm thickness	Flake	[64]
Chemical synthesis	Zinc Nitrate hexahydrate, Ammonia solution	0.66–0.92 µm	Ellipsoid	[65]

**Table 2:** Synthesis of ZnO nanoparticles via microfluidics.

Materials	Microfluidic Device	Nanoparticles Size	Nanoparticles Shape	Reference
Zinc nitrate hexahydrate, Hexamethylenetetramine (HTMA), Polyethylenimine (PEI)	Cr-ZnO seeded PDMS plate	2–3 µm	Wire	[71]
Zinc nitrate, Hexamethylenetetramine	Glass/silicone plate	1–3 µm	Wire	[72]
Zinc acetate dihydrate, Benzyl alcohol	PDMS, Aluminium mounting	150 nm	Sphere	[45]
Zn(NO_3_)_2_, NaOH	Micro T-mixer	3–4 µm	Flower	[70]
Zinc acetate, Ammonium acetate, Sodium hydroxide	Micro T-mixer	347 nm thickness	Flower	[73]
Zinc nitrate, NaOH	Spiral microchannel PDMS	100 nm	Sphere, ellipsoid, rod, cube	[18]
Zinc acetate dihydrate, Zinc nitrate hexahydrate, Hexamethilenetetramine, Ammonium hydroxide	Straight microchannel PDMS	0.16 µm diameter, 8.17 µm length	Wire	[74]
Zinc nitrate hexahydrate, Sodium hydrate, Sodium Borohydrate	Polytetrafluoroethylene	500 nm	Hedgehog	[75]

## Data Availability

Not applicable.

## References

[1] Behnaz M., Kasraei S., Yadegari Z., Zare F., Nahvi G. (2021). Effects of Orthodontic Bonding Containing TiO_2_ and ZnO Nanoparticles on Prevention of White Spot Lesions: An In Vitro Study. Biointerface Res. Appl. Chem..

[2] Darshitha M.N. (2021). Richa Sood. Review on Synthesis and Applications of Zinc Oxide Nanoparticles. Preprints.

[3] Sadighian S., Sharifan K., Khanmohammadi A., Rohani M.K. (2022). A Facile Synthesis of Fe3O4@SiO2@ZnO for Curcumin Delivery. Biointerface Res. Appl. Chem..

[4] Gerbreders V., Krasovska M., Sledevskis E., Gerbreders A., Mihailova I., Tamanis E., Ogurcovs A. (2020). Hydrothermal Synthesis of ZnO Nanostructures with Controllable Morphology Change. CrystEngComm.

[5] Lghazi Y., Bahar J., Youbi B., Himi M.A., Elhaimer C., Elouadrhiri A., Bimaghra I., Ouknin M., Majidi L. (2022). Nucleation/Growth and Optical Proprieties of Co-doped ZnO Electrodeposited on ITO Substrate. Biointerface Res. Appl. Chem..

[6] Droepenu E.K., Asare E.A., Dampare S.B., Adotey D.K., Gyampoh A.O., Kumi-Arhin E. (2021). Laboratory and Commercial Synthesized Zinc Oxide Nanoparticles Adsorption onto Coconut Husk: Characterization, Isotherm, Kinetic, and Thermodynamic Studies. Biointerface Res. Appl. Chem..

[7] Morkoc H., Özgür Ü. (2009). Zinc Oxide: Fundamentals, Materials and Device Technology.

[8] Droepenu E.K., Wee B.S., Chin S.F., Kok K.Y., Maligan M.F. (2022). Zinc Oxide Nanoparticles Synthesis Methods and Its Effect on Morphology: A Review. Biointerface Res. Appl. Chem..

[9] Geurts J. (2010). Crystal Structure, Chemical Binding, and Lattice Properties. Springer Ser. Mater. Sci..

[10] Liu S., Mannsfeld S.C.B., Wang W.M., Sun Y.S., Stoltenberg R.M., Bao Z. (2009). Patterning of α-Sexithiophene Single Crystals with Precisely Controlled Sizes and Shapes. Chem. Mater..

[11] Tian L., Huang Z., Na W., Liu Y., Wang S., He Y., Cheng W., Huang T.-Z., Li Z., Li T. (2022). The Heterojunction MnO_2_ Nanosheet Decorated Ag Nanowires with Enhanced Oxidase-like Activity for Sensitively Dual-Mode Detection of Glutathione. Nanoscale.

[12] Bao Y., Yan Y., Ma J., Zhang W., Zong Y. (2020). ZnO Encapsulants: Design and New View. Adv. Colloid Interface Sci..

[13] Borysiewicz M.A. (2019). ZnO as a Functional Material, a Review. Crystals.

[14] Hira I., Kumari R., Saini A.K., Gullilat H., Saini V., Sharma A.K., Saini R.v. (2022). Apoptotic Cell Death Induction through Pectin, Guar Gum and Zinc Oxide Nanocomposite in A549 Lung Adenocarcinomas. Biointerface Res. Appl. Chem..

[15] Noman M.T., Amor N., Petru M. (2021). Synthesis and Applications of ZnO Nanostructures (ZONSs): A Review. Crit. Rev. Solid State Mater. Sci..

[16] Whitesides G.M. (2006). The Origins and the Future of Microfluidics. Nature.

[17] Convery N., Gadegaard N. (2019). 30 Years of Microfluidics. Micro Nano Eng..

[18] Hao N., Zhang M., Zhang J.X.J. (2020). Microfluidics for ZnO Micro-/Nanomaterials Development: Rational Design, Controllable Synthesis, and on-Chip Bioapplications. Biomater. Sci..

[19] Han Z., Jiang X., Jiang X., Bai C., Liu M. (2019). Microfluidic Synthesis of Functional Nanoparticles. Nanotechnology and Microfluidics.

[20] Shepherd S.J., Issadore D., Mitchell M.J. (2021). Microfluidic Formulation of Nanoparticles for Biomedical Applications. Biomaterials.

[21] Tian F., Cai L., Liu C., Sun J. (2022). Microfluidic technologies for nanoparticle formation. Lab Chip.

[22] Amreen K., Goel S. (2021). Review—Miniaturized and Microfluidic Devices for Automated Nanoparticle Synthesis. ECS J. Solid State Sci. Technol..

[23] Sounart T.L., Safier P.A., Voigt J.A., Hoyt J., Tallant D.R., Matzke C.M., Michalske T.A. (2007). Spatially-Resolved Analysis of Nanoparticle Nucleation and Growth in a Microfluidic Reactor. Lab Chip.

[24] Abedini-nassab R., Pouryosef Miandoab M., Şaşmaz M. (2021). Microfluidic Synthesis, Control, and Sensing of Magnetic Nanoparticles: A Review. Micromachines.

[25] Tang S.Y., Qiao R., Yan S., Yuan D., Zhao Q., Yun G., Davis T.P., Li W. (2018). Microfluidic Mass Production of Stabilized and Stealthy Liquid Metal Nanoparticles. Small.

[26] Baig N., Kammakakam I., Falath W., Kammakakam I. (2021). Nanomaterials: A Review of Synthesis Methods, Properties, Recent Progress, and Challenges. Mater. Adv..

[27] Abdel-Baset T.A., Belhaj M. (2021). Structural Characterization, Dielectric Properties and Electrical Conductivity of ZnO Nanoparticles Synthesized by Co-Precipitation Route. Phys. B Condens. Matter..

[28] Mohan S., Vellakkat M., Aravind A., Reka U. (2020). Hydrothermal Synthesis and Characterization of Zinc Oxide Nanoparticles of Various Shapes under Different Reaction Conditions. Nano Express.

[29] Aneesh P.M., Vanaja K.A., Jayaraj M.K. (2007). Synthesis of ZnO Nanoparticles by Hydrothermal Method. Nanophotonic Mater. IV.

[30] Ong C.B., Ng L.Y., Mohammad A.W. (2018). A Review of ZnO Nanoparticles as Solar Photocatalysts: Synthesis, Mechanisms and Applications. Renew. Sustain. Energy Rev..

[31] Mirzaeifard Z., Shariatinia Z., Jourshabani M., Rezaei Darvishi S.M. (2020). ZnO Photocatalyst Revisited: Effective Photocatalytic Degradation of Emerging Contaminants Using S-Doped ZnO Nanoparticles under Visible Light Radiation. Ind. Eng. Chem. Res..

[32] Mintcheva N., Aljulaih A.A., Wunderlich W., Kulinich S.A., Iwamori S. (2018). Laser-Ablated ZnO Nanoparticles and Their Photocatalytic Activity toward Organic Pollutants. Materials.

[33] Al Abdullah K., Awad S., Zaraket J., Salame C. (2017). Synthesis of ZnO Nanopowders by Using Sol-Gel and Studying Their Structural and Electrical Properties at Different Temperature. Energy Procedia.

[34] Hasnidawani J.N., Azlina H.N., Norita H., Bonnia N.N., Ratim S., Ali E.S. (2016). Synthesis of ZnO Nanostructures Using Sol-Gel Method. Procedia Chem..

[35] Tiplea R.E., Lemnaru G.M., Trușcă R.D., Holban A., Kaya M.G.A., Dragu L.D., Ficai D., Ficai A., Bleotu C. (2021). Antimicrobial Films Based on Chitosan, Collagen, and Zno for Skin Tissue Regeneration. Biointerface Res. Appl. Chem..

[36] Mahmood N.B., Saeed F.R., Gbashi K.R., Mahmood U.S. (2022). Synthesis and Characterization of Zinc Oxide Nanoparticles via Oxalate Co-Precipitation Method. Mater. Lett. X.

[37] Müller R., Huber F., Gelme O., Madel M., Scholz J.P., Minkow A., Herr U., Thonke K. (2019). Chemical Vapor Deposition Growth of Zinc Oxide on Sapphire with Methane: Initial Crystal Formation Process. Cryst. Growth Des..

[38] Reuge N., Bacsa R., Serp P., Caussat B. (2009). Chemical Vapor Synthesis of Zinc Oxide Nanoparticles: Experimental and Preliminary Modeling Studies. J. Phys. Chem. C.

[39] Hung L.-H., Lee A.P., Engineering B. (2007). Microfluidic devices for the synthesis of nanoparticles and biomaterials. J. Med. Biol. Eng..

[40] Bressan L.P., Robles-Najar J., Adamo C.B., Quero R.F., Costa B.M.C., de Jesus D.P., da Silva J.A.F. (2019). 3D-Printed Microfluidic Device for the Synthesis of Silver and Gold Nanoparticles. Microchem. J..

[41] Thanh N.T.K., Maclean N., Mahiddine S. (2014). Mechanisms of Nucleation and Growth of Nanoparticles in Solution. Chem. Rev..

[42] Tofighi G., Lichtenberg H., Gaur A., Wang W., Wild S., Herrera Delgado K., Pitter S., Dittmeyer R., Grunwaldt J.D., Doronkin D.E. (2022). Continuous Synthesis of Cu/ZnO/Al_2_O_3_ nanoparticles in a Co-Precipitation Reaction Using a Silicon Based Microfluidic Reactor. React. Chem. Eng..

[43] Shaba E.Y., Jacob J.O., Tijani J.O., Suleiman M.A.T. (2021). A Critical Review of Synthesis Parameters Affecting the Properties of Zinc Oxide Nanoparticle and Its Application in Wastewater Treatment. Appl. Water Sci..

[44] Niculescu A.G., Chircov C., Bîrcă A.C., Grumezescu A.M. (2021). Nanomaterials Synthesis through Microfluidic Methods: An Updated Overview. Nanomaterials.

[45] Stolzenburg P., Lorenz T., Dietzel A., Garnweitner G. (2018). Microfluidic Synthesis of Metal Oxide Nanoparticles via the Nonaqueous Method. Chem. Eng. Sci..

[46] Shrimal P., Jadeja G., Patel S. (2020). A Review on Novel Methodologies for Drug Nanoparticle Preparation: Microfluidic Approach. Chem. Eng. Res. Des..

[47] Li Z., Mak S.Y., Sauret A., Shum H.C. (2014). Syringe-Pump-Induced Fluctuation in All-Aqueous Microfluidic System Implications for Flow Rate Accuracy. Lab Chip.

[48] Zhang X., Chen Z., Huang Y. (2015). A Valve-Less Microfluidic Peristaltic Pumping Method. Biomicrofluidics.

[49] Södergren S., Svensson K., Hjort K. (2021). Microfluidic Active Pressure and Flow Stabiliser. Sci. Rep..

[50] Sauret A., Spandagos C., Shum H.C. (2012). Fluctuation-induced dynamics of multiphase liquid jets with ultra-low interfacial tension. Lab Chip.

[51] Zhai J., Li H., Wong A.H.H., Dong C., Yi S., Jia Y., Mak P.I., Deng C.X., Martins R.P. (2020). A Digital Microfluidic System with 3D Microstructures for Single-Cell Culture. Microsyst. Nanoeng..

[52] Capretto L., Cheng W., Hill M., Zhang X. (2011). Micromixing within Microfluidic Devices. Top Curr. Chem..

[53] Lee C.Y., Chang C.L., Wang Y.N., Fu L.M. (2011). Microfluidic Mixing: A Review. Int. J. Mol. Sci..

[54] Bayareh M., Ashani M.N., Usefian A. (2020). Active and Passive Micromixers: A Comprehensive Review. Chem. Eng. Process. Process Intensif..

[55] Liao Y., Mechulam Y., Lassalle-Kaiser B. (2021). A Millisecond Passive Micromixer with Low Flow Rate, Low Sample Consumption and Easy Fabrication. Sci. Rep..

[56] Vijayanandh V., Pradeep A., Suneesh P.V., Satheesh Babu T.G. (2019). Design and Simulation of Passive Micromixers with Ridges for Enhanced Efficiency. IOP Conf. Ser. Mater. Sci. Eng..

[57] Raza W., Hossain S., Kim K.Y. (2020). A Review of Passive Micromixers with a Comparative Analysis. Micromachines.

[58] Tan J.N., Neild A. (2012). Microfluidic Mixing in a Y-Junction Open Channel. AIP Adv..

[59] Tello A., Boulett A., Sánchez J., Pizarro G.d.C., Soto C., Linarez Pérez R., Sanhueza R., Oyarzún D.P. (2021). An Unexplored Strategy for Synthesis of ZnO Nanowire Films by Electrochemical Anodization Using an Organic-Based Electrolyte. Morphological and Optical Properties Characterization. Chem. Phys. Lett..

[60] Tian B., Liu S., Zhang Y., Li C., Wang Z. (2017). Hydrophilic, Mesoporous Structural ZnO Nanospheres for PH-Triggered Release of Drug. Mater. Lett..

[61] Zhang Y., Chung J., Lee J., Myoung J., Lim S. (2011). Synthesis of ZnO Nanospheres with Uniform Nanopores by a Hydrothermal Process. J. Phys. Chem. Solids.

[62] Qu Y., Huang R., Qi W., Shi M., Su R., He Z. (2020). Controllable Synthesis of ZnO Nanoflowers with Structure-Dependent Photocatalytic Activity. Catal. Today.

[63] Tripathi R.M., Bhadwal A.S., Gupta R.K., Singh P., Shrivastav A., Shrivastav B.R. (2014). ZnO Nanoflowers: Novel Biogenic Synthesis and Enhanced Photocatalytic Activity. J. Photochem. Photobiol. B.

[64] Samanta P.K., Saha A. (2015). Wet Chemical Synthesis of ZnO Nanoflakes and Photoluminescence. Optik.

[65] Pu X., Zhang D., Yi X., Shao X., Li W., Sun M., Li L., Qian X. (2010). Rapid Chemical Synthesis and Optical Properties of ZnO Ellipsoidal Nanostructures. Adv. Powder Technol..

[66] Lee B.J., Jo S.-I., Heo S.G., Lee W.Y., Jeong G.H. (2021). Structure-Controllable Synthesis of ZnO Nanowires Using Water Vapor in an Atmospheric-Pressure Microwave Plasma System. Curr. Appl. Phys..

[67] Li S., Gross G.A., Günther P.M., Köhler J.M. (2011). Hydrothermal Micro Continuous-Flow Synthesis of Spherical, Cylinder-, Star- and Flower-like ZnO Microparticles. Chem. Eng. J..

[68] Duan J.X., Wang H., Huang X.T. (2007). Synthesis and Characterization of ZnO Ellipsoid-like Nanostructures. Chin. J. Chem. Phys..

[69] Hao N., Xu Z., Nie Y., Jin C., Closson A.B., Zhang M., Zhang J.X.J. (2019). Microfluidics-Enabled Rational Design of ZnO Micro-/Nanoparticles with Enhanced Photocatalysis, Cytotoxicity, and Piezoelectric Properties. Chem. Eng. J..

[70] Choi C.H., Chang C.H. (2014). Aqueous Synthesis of Tailored ZnO Nanocrystals, Nanocrystal Assemblies, and Nanostructured Films by Physical Means Enabled by a Continuous Flow Microreactor. Cryst. Growth Des..

[71] Kim J., Hong J.W., Kim D.P., Shin J.H., Park I. (2012). Nanowire-Integrated Microfluidic Devices for Facile and Reagent-Free Mechanical Cell Lysis. Lab Chip.

[72] McPeak K.M., Baxter J.B. (2009). ZnO Nanowires Grown by Chemical Bath Deposition in a Continuous Flow Microreactor. Cryst. Growth Des..

[73] Kim K.J., Kreider P.B., Choi C., Chang C.H., Ahn H.G. (2013). Visible-Light-Sensitive Na-Doped p-Type Flower-like ZnO Photocatalysts Synthesized via a Continuous Flow Microreactor. RSC Adv..

[74] Guo L., Shi Y., Liu X., Han Z., Zhao Z., Chen Y., Xie W., Li X. (2018). Enhanced Fluorescence Detection of Proteins Using ZnO Nanowires Integrated inside Microfluidic Chips. Biosens. Bioelectron..

[75] Tao S., Yang M., Chen H., Ren M., Chen G. (2016). Continuous Synthesis of Hedgehog-like Ag-ZnO Nanoparticles in a Two-Stage Microfluidic System. RSC Adv..

[76] Baruah A., Jindal A., Acharya C., Prakash B., Basu S., Ganguli A.K. (2017). Microfluidic reactors for the morphology controlled synthesis and photocatalytic study of ZnO nanostructures. J. Micromech. Microeng..

[77] Zhao C., Zhang J., Hu Y., Robertson N., Hu P.A., Child D., Gibson D., Fu Y.Q. (2015). In-Situ Microfluidic Controlled, Low Temperature Hydrothermal Growth of Nanoflakes for Dye-Sensitized Solar Cells. Sci. Rep..

[78] Choi C.H., Su Y.W., Chang C.H. (2013). Effects of Fluid Flow on the Growth and Assembly of ZnO Nanocrystals in a Continuous Flow Microreactor. CrystEngComm.

[79] Wibowo A., Marsudi M.A., Amal M.I., Ananda M.B., Stephanie R., Ardy H., Diguna L.J. (2020). ZnO Nanostructured Materials for Emerging Solar Cell Applications. RSC Adv..

[80] Kamaruzaman D., Ahmad N., Rosly M.A., Mamat M.H. (2021). Piezoelectric Energy Harvesting Based on ZnO: A Review. AIP Conf. Proc..

[81] Sathishkumar P., Li Z., Govindan R., Jayakumar R., Wang C., Long Gu F. (2021). Zinc Oxide-Quercetin Nanocomposite as a Smart Nano-Drug Delivery System: Molecular-Level Interaction Studies. Appl. Surf. Sci..

[82] Naghizadeh A., Mohammadi-Aghdam S., Mortazavi-Derazkola S. (2020). Novel CoFe_2_O_4_@ZnO-CeO_2_ Ternary Nanocomposite: Sonochemical Green Synthesis Using Crataegus Microphylla Extract, Characterization and Their Application in Catalytic and Antibacterial Activities. Bioorg. Chem..

[83] Pagaduan J.V., Sahore V., Woolley A.T. (2015). Applications of microfluidics and microchip electrophoresis for potential clinical biomarker analysis. Anal. Bioanal. Chem..

[84] Dumontel B., Susa F., Limongi T., Canta M., Racca L., Chiodoni A., Garino N., Chiabotto G., Centomo M.L., Pignochino Y. (2019). ZnO Nanocrystals Shuttled by Extracellular Vesicles as Effective Trojan Nano-Horses against Cancer Cells. Nanomedicine.

[85] Radwan-Pragłowska J., Janus Ł., Piątkowski M., Sierakowska A., Matysek D. (2020). ZnO Nanorods Functionalized with Chitosan Hydrogels Crosslinked with Azelaic Acid for Transdermal Drug Delivery. Colloids Surf. B Biointerfaces.

[86] Laurenti M., Cauda V. (2017). ZnO Nanostructures for Tissue Engineering Applications. Nanomaterials.

[87] Coluccio M.L., Perozziello G., Malara N., Parrotta E., Zhang P., Gentile F., Limongi T., Raj P.M., Cuda G., Candeloro P. (2019). Microfluidic Platforms for Cell Cultures and Investigations. Microelectron. Eng..

[88] Kung C.T., Gao H., Lee C.Y., Wang Y.N., Dong W., Ko C.H., Wang G., Fu L.M. (2020). Microfluidic Synthesis Control Technology and Its Application in Drug Delivery, Bioimaging, Biosensing, Environmental Analysis and Cell Analysis. Chem. Eng. J..

[89] Sochol R.D., Sweet E., Glick C.C., Wu S.Y., Yang C., Restaino M., Lin L. (2018). 3D Printed Microfluidics and Microelectronics. Microelectron. Eng..

[90] Wang J., Mountziaris T.J. (2013). Homogeneous immunoassays based on fluorescence emission intensity variations of zinc selenide quantum dot sensors. Biosens. Bioelectron..

[91] Schlücker S. (2009). SERS microscopy: Nanoparticle probes and biomedical applications. Chemphyschem A Eur. J. Chem. Phys. Phys. Chem..

[92] Kumar A.R., Shanmugasundaram K.B., Li J., Zhang Z., Ibn Sina A.A., Wuethrich A., Trau M. (2020). Ultrasensitive Melanoma Biomarker Detection Using a Microchip SERS Immunoassay with Anisotropic Au-Ag Alloy Nanoboxes. RSC Adv..

[93] Xie Y., Yang S., Mao Z., Li P., Zhao C., Cohick Z., Huang P.-H., Huang T.J. (2014). In Situ Fabrication of 3D Ag @ ZnO Nanostructures for Microfluidic. ACS Nano.

[94] Li S., Gao Y., Chen X., Qin L., Cheng B., Wang S., Wang S., Zhao G., Liu K., Zhang N. (2017). Highly Efficient Isolation and Release of Circulating Tumor Cells Based on Size-Dependent Filtration and Degradable ZnO Nanorods Substrate in a Wedge-Shaped Microfluidic Chip. Biomed. Microdevices.

[95] Han Z., Li J., He W., Li S., Li Z., Chu J., Chen Y. (2013). A Microfluidic Device with Integrated ZnO Nanowires for Photodegradation Studies of Methylene Blue under Different Conditions. Microelectron. Eng..

[96] Mohamad I.S., Norizan M.N., Mahmed N., Jamalullail N., Halin D.S.C., Salleh M.A.A.M., Sandu A.V., Baltatu M.S., Vizureanu P. (2022). Enhancement of Power Conversion Efficiency with Zinc Oxide as Photoanode and Cyanococcus, Punica Granatum L., and Vitis Vinifera as Natural Fruit Dyes for Dye-Sensitized Solar Cells. Coatings.

[97] Nielsen A.V., Beauchamp M.J., Nordin G.P., Woolley A.T. (2020). 3D Printed Microfluidics. Annu. Rev. Anal. Chem..

